# MIAT inhibits proliferation of cervical cancer cells through regulating miR-150-5p

**DOI:** 10.1186/s12935-020-01338-0

**Published:** 2020-06-15

**Authors:** Yanbin Liu, Xingzhi Li, Hui Zhang, Yali Huang

**Affiliations:** 1 Institute of Immunology and Molecular Medicine, Jining Medical University, Jining, Shandong Province China; 2 Department of Urological Surgery, Longgang District People’s Hospital of Shenzhen, Shenzhen, China; 3 Department of Biochemistry, Zhongshan School of Medicine, Sun Yat-sen University, Guangzhou, China

**Keywords:** MIAT, miR-150-5p, CDKN1B, Cervical cancer, Long non-coding RNA

## Abstract

**Background:**

To characterize the MIAT expression in cervical cancer and elucidate its mechanistic involvement in the tumor biology of this disease.

**Methods:**

The relative expression of MIAT and miR-150 was determined by real-time PCR. Cell proliferation was measured by the CCK-8 and clonogenic assay. The anchorage-independent growth was evaluated by soft agar assay. The in vivo tumor progression was assayed with xenograft mice model. The regulatory effect of miR-150 on MIAT was interrogated by luciferase reporter assay. The endogenous CNKD1B protein was detected by western blotting.

**Results:**

The low expression of MIAT was characterized in cervical cancer, which associated with relatively poor prognosis. Ectopic expression of MIAT inhibited malignant growth of cervical cancer both in vitro and in vivo. Mechanistically, MIAT regulated CDKN1B expression via competition with miR-150, and miR-150-inhibition directly suppressed cervical cancer cell growth.

**Conclusions:**

Our study characterized the anti-tumor property of MIAT in cervical cancer and elucidated its competitively regulation of CDKN1B with miR-150. Our data highlighted the critical role of MIAT-miR-150-CDKN1B signaling axis in cervical cancer.

## Background

Cervical cancer is both the fourth-most common cause of cancer and the fourth-most common cause of death from gynecologic cancer. It’s estimated that 528,000 new cases were diagnosed and 266,000 deaths were claim by this disease worldwide in 2012 [1]. Epidemiologic investigation reveals that more than 90% of morbidity are caused by human papillomavirus infection [2]. Histologically, most of cervical carcinoma are categorized into either squamous cell carcinomas (90%) and adenocarcinoma (10%). The preventive vaccines against human papillomavirus infection may control up to 90% occurrence of cervical cancers [3]. The mainstay clinical treatments of cervical cancer consist of some combination of surgery, chemotherapy and radiotherapy [4]. However, the deep insights into the fundamental molecular events underlying this disease are still in urgent need to contribute to our comprehensive understanding into etiology of cervical cancer.

Long non-coding RNAs (lncRNAs) are group of RNAs with average length more than 200 nucleotides and without protein-coding potential, which account for about 80% of all known human transcripts [5]. The diverse biological functions of lncRNAs are increasingly acknowledged involving in the complex gene regulatory network. Most importantly, lncRNAs play critical roles in various facets of post-transcriptional processing of mRNA, and significantly influence pre-mRNA splicing, transportation, translation and degradation [6]. Among which, Myocardial Infarction Associated Transcript (MIAT) encodes a spliced lncRNA that may constitute a component of nuclear matrix, and altered expression of this molecule is reported to be associated with susceptibility to myocardial infarction [7]. Another common human disease relates to deficiency in this gene was schizophrenia [8]. More recently, emerging evidences suggest the mechanistic involvements of MIAT in diverse human malignancies as well [9–11]. However, the expression pattern and involvements of MIAT in cervical cancer are not fully investigated. Here we set out to determine the expression status of MIAT and seek to elucidate its mechanistic linkage to cervical cancer.

## Materials and methods

### Patient samples

The human related study was approved and authorized by the Institutional Ethics Committee. In total, 21 cervical carcinoma tumors and adjacent normal tissues were collected between 2016 to 2019, and the written informed consents were obtained. None of enrolled patients have received prior treatments before surgery. The cervical carcinoma was histologically validated by three independent experienced pathologists. Fresh tissue samples were flash frozen in liquid nitrogen for preservative purpose.

### Cell culture

The immortalized human cervical epithelial cell line H8 and cervical carcinoma cell lines C-33 A, Ca Ski, HeLa, SiHa and HT-3 were obtained from and authenticated by the America Typical Culture Collection (ATCC, VA, USA). All cells were maintained in RPMI-1640 medium (Gibco, CA, USA) supplemented with 10% fetal bovine serum (Hyclone, MA, USA) and 1% P–S–G (penicillin–streptomycin–glutamine, Gibco, CA, USA). The log phase cells were cultured at 37 °C in humidified incubator with 5% CO_2_.

### Transfection

Transfection was performed with Lipofectamine 2000 (Invitrogen, CA, USA) according to the manufacturer’s recommendation. Briefly, HeLa or HT-3 cells at log phase were seeded into 6-well plate (5 × 10^5^ cells/well). The indicated plasmids (1 μg) were packaged with Lipofectamine 2000 (10 μl) homogenously at room temperature for 5 min and added into each well. The parallel GFP transfection was performed for 24 h to evaluate transfection efficiency with inverted fluorescence microscope (Nikon, CA, USA). shMIAT: 5′-CCAGGCUCCUUUAAACCAATT-3′.

### Real-time PCR

The RNA was extracted from either tissue samples or cell lines with TRIzol reagent (Invitrogen, CA, USA). The quality and quantity of RNA was determined by the BioAnalyzer 2100 (Agilent, CA, USA). The cDNA was prepared by reverse transcription of equal amount of RNA (1 μg) using the PrimeScript First Strand cDNA Synthesis Kit (Clontech, MO, USA). The cDNA product was then 1: 5 diluted with ultra-pure water and 1 μl was used for real-time PCR determination. The real-time PCR was performed on ABI Prism 7900 HT. All primers were available upon request. The primer sequences were listed as follows [12]: MIAT Forward 5′-TCTTCATGTCAGAACACGCTTTA-3′, reverse 5′-AAGGTCACCCGAGGTCCAA-3′; CDKN1B Forward 5′-TAATTGGGGCTCCGGCTAACT-3′, reverse 5′-TGCAGGTCGCTTCCTTATTCC-3′; GAPDH Forward 5′-GGAGCGAGATCCCTCCAAAAT-3′, reverse 5′-GGCTGTTGTCATACTTCTCATGG-3′; MiR-150-5p Forward 5′-TGCGGTCTCCCAACCCTTG-3′, reverse 5′-CCAGTGCAGGGTCCGAGGT-3′; U6 Forward 5′-TGCGGGTGCTCGCTTCGGCAGC-3′, reverse 5′-CCAGTGCAGGGTCCGAGGT.

### Dual-luciferase reporter assay

The full length of MIAT transcript (either wild-type or miR-150-5p target region mutation) was fused to luciferase into pGL4 plasmid, and co-transfected HeLa or HT-3 cells with miR-150-5p. Cells were harvested 48 h later and relative luciferase activities were measured with Dual Luciferase Assay System (Promega, WI, USA) [13].

### Western blots

The cell lysates were prepared in RIPA lysis buffer and protein concentration was measured using BCA Protein Assay Kit (Pierce, MA, USA). Protein (20 μg) was resolved by SDS-PAGE and transferred onto PVDF membrane on ice. After brief blocking with 5% milk, the PVDF membrane was incubated with specific primary antibodies (rabbit anti-CDKN1B, HPA059086, 1:1000; rabbit anti-β-actin, SAB5500001,1:1000; Sigma-Aldrich, MO, USA) at 4 °C overnight. The membrane was washed with TBST (0.05% Tween-20, Sigma, MO, USA) and hybridized with HRP-labeled secondary antibody (goat anti-rabbit, #7074, 1:5000; Cell Signaling Technology) at room temperature for another hour. The protein blots were visualized using enhanced chemiluminescence reagent (ECL, Cwbiotech, Beijing, China) [14].

### Cell proliferation assay

The cell proliferation was measured using Cell Counting Kit-8 (CCK-8, Dojindo, Dalian, China). The indicated cells were seeded into 96-well plate (10^4^/well) in triplicate for 24-h culture and subjected to the specified treatments (EV or MIAT). The CCK-8 solution (10 μL) was subsequently added into each well and allowed for 1-h incubation at 37 °C. The absorption at 450 nm was recorded on microplate reader (Molecular Devices, CA, USA) and relative cell count was calculated [15].

### Colony formation assay

The indicated cells were placed into 6-well plate (400 cells per well) and transfected with either empty vectors or MIAT-expressing plasmids for 24 h. The fresh medium was replaced and followed by consecutive culture for 2 weeks. The resultant colonies were fixed with 4% PFA and stained with 0.25% crystal violet for visualization. The representative images were captured under light microscopy [16].

### Soft agar assay

HeLa and HT-3 cells were transfected with indicated plasmids and allowed for 24 h culture. The single cell suspension was prepared in 2 × RPMI 1640 via trypsin digestion and mixed with equal volume of 0.7% low-melting-point agarose (Sigma, MO, USA). The mixture was layered on top of solidified 0.7% agarose (in serum-free growth medium) and allowed for consecutive culture up to 2 weeks. The colonies with more than 50 cells were visualized by 0.05% crystal violet staining and counted under light microscope [17].

### Xenograft tumor

The BALB/c-nude mice (female, 20–22 g) were purchased from the VitalRiver (Beijing, China), and randomly divided into control and MIAT groups after 1-week acclimatization. The single-cell resuspension of indicated cell (10^6^ cells infected with both E.V and MIAT lentivirus) was mixed with equal volume of Matrigel (BD Biosciences, CA, USA) on ice, and subcutaneously inoculated into the lower flank of immunodeficiency mice. Tumor growth was monitored regularly and volume was estimated using digital caliper according to the formula: TV (mm^3^) = length × width^2^/2. The animal study was conducted in pathogen-free environment and the protocol was approved by the Institutional Committee of Animal Care and Use [18].

### Statistical analysis

All data were obtained from at least three independent experiments. Data was analyzed using PRISM 7.0 software and presented as Mean ± standard deviation (SD). The one-way analysis of variance (ANOVA) followed by *t* test was employed for statistical comparison. The *p* value was calculated and *p* < 0.05 was considered as statistically different.

## Results

### Down-regulation of MIAT in human cervical carcinoma

**Fig. 1:**
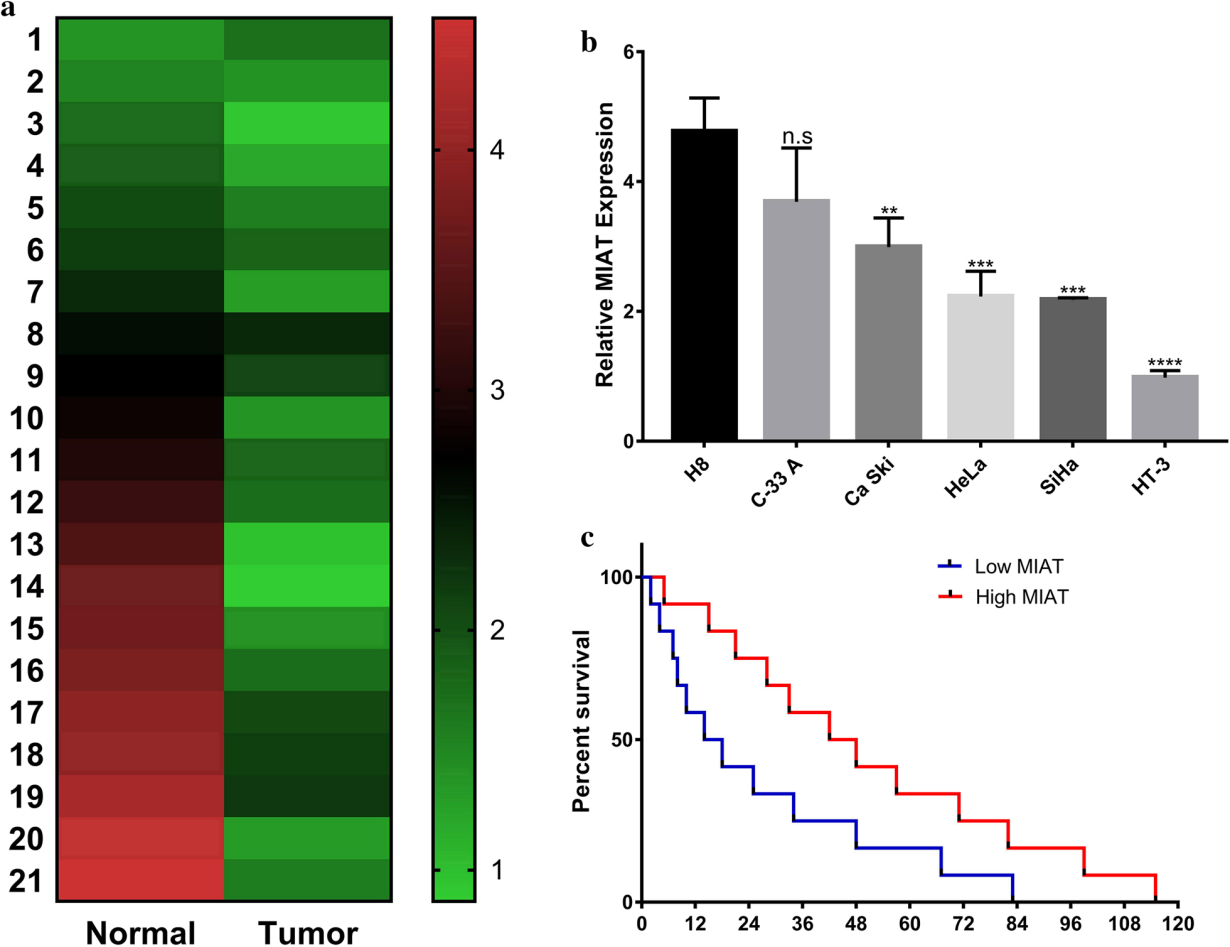
Low expression of MIAT in human cervical cancer. **a** The expression of MIAT was determined by real-time PCR and normalized to β-actin in human cervical cancer samples (n = 21 pairs); **b** Relative expression of MIAT was measured by real-time PCR in human cervical cancer cell line panel (n = 5) in comparison with immortalized human cervical epithelial cell. n.s: no significance, ** *p* < 0.01, *** *p* < 0.001, ****p < 0.0001; **c** Kaplan–Meier curve of cumulative survival in cervical cancer patients with high MIAT (n = 12) and low MIAT expression (n = 12)

First, the relative expression of MIAT in cervical carcinoma was determined by q-PCR in 21 pairs of cervical tumors and adjacent benign tissues. As shown in Fig. 1a, MIAT was significantly lower in most of cervical tumors in comparison with normal control, which indicated the potential anti-tumor properties of MIAT in this disease. Low MIAT abundance significantly associated with tumor progression, stage and metastasis as well (Table 1). We further consolidated this preliminary observation in panel of cervical cancer cell lines and the immortalized human cervical epithelial cell H8. Consistent with the results obtained from clinical samples, MIAT transcripts were down-regulated in cancer cell lines compared to H8 cells (Fig. 1b). The HeLa and HT-3 cells were shown with prominently low levels of MIAT, which served as appropriate candidates for the following investigations with ectopic over-expression. Moreover, Kaplan–Meier cumulative survival curve exhibited more favorable outcome in MIAT-high cervical cancer group (with median survival time 42 months v.s 15 months in MIAT-low group, Fig. 1c), which consolidated the tumor suppressor role of MIAT in vivo.

**Table 1:** Correlation between the clinicopathological features and MIAT level

Variable	Number of cases	MIAT		
		High	Low	p value
	24	12	12	
Age (years)				0.6968
< 60	10	6	4	
> 60	14	7	7	
Tumor size				0.0324*
< 5 cm	8	6	2	
≥ 5 cm	16	4	12	
Tumor stage				0.0111*
T1, T2	11	8	3	
T3, T4	13	2	11	
Lymphatic metastasis				0.0145*
Negative	5	4	1	
Positive	19	3	16	

*indicated statistical significance

### MIAT-overexpression inhibited malignant growth in cervical cancer cells

**Fig. 2:**
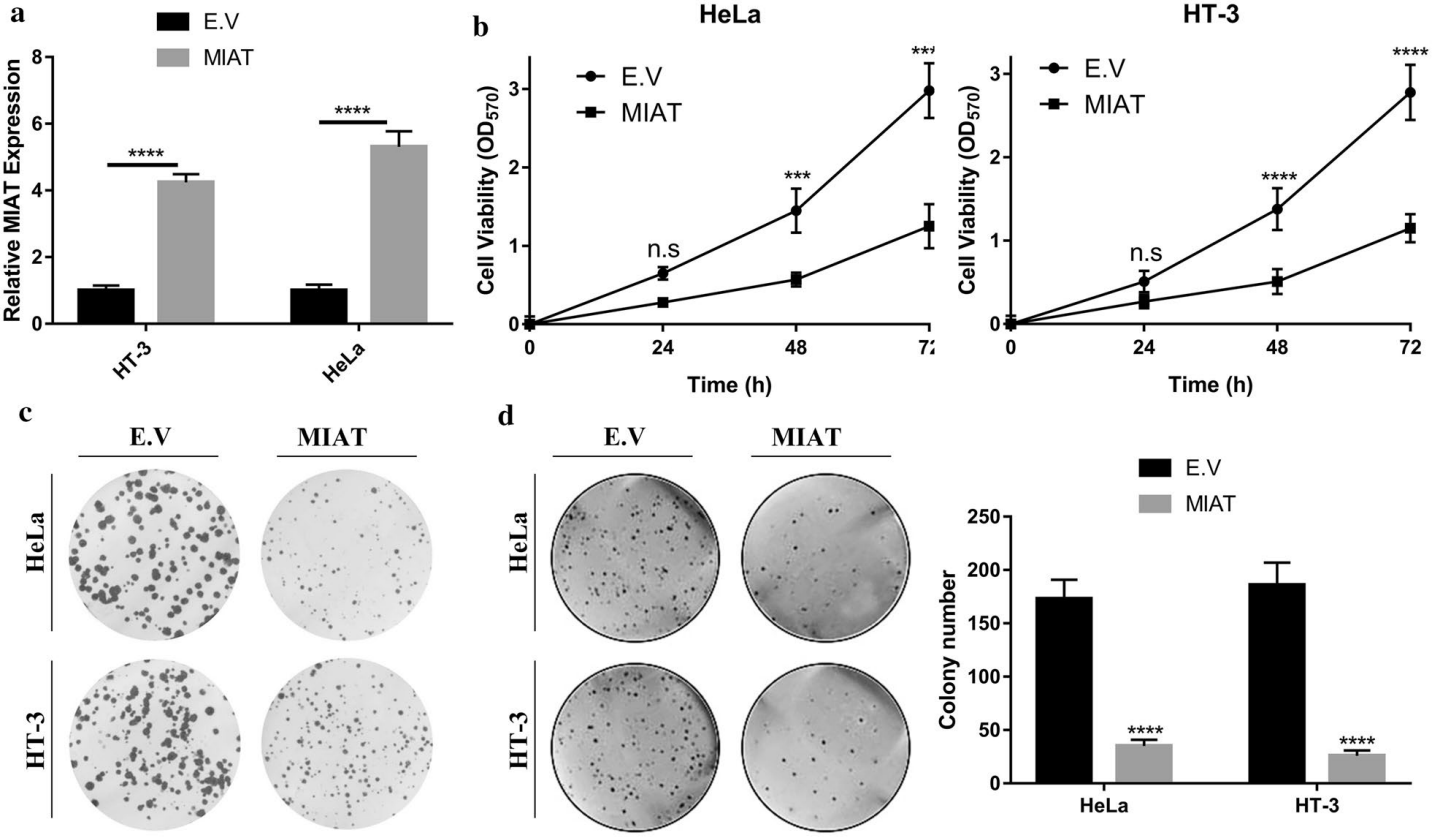
Ectopic expression of MIAT inhibited malignant progression in cervical cancer cells. **a** HeLa and HT-3 cells were transfected with either empty vector (E.V) or MIAT plasmid using lipofectamine 2000. The expression efficiency was measured by real-time PCR at 24 h post-transfection. ****p < 0.0001; **b** Cell viability was determined in MIAT-proficient HeLa and HT-3 cells with CCK-8 kit. n.s: no significance, *** *p* < 0.001, ****p < 0.0001; **c** Cell proliferation was measured by 1-week clonogenic assay. The representative image was acquired under optical microscope with crystal violet staining. **d** The anchorage-independent growth of both MIAT-proficient and naïve HeLa and HT-3 cells was evaluated with soft agar assay (left pane), the corresponding statistical results from three individual fields were shown in the right pane, ****p < 0.0001

Next, we attempted to experimentally uncover the tumor suppressive properties of MIAT in cervical cancer cell lines. The ectopic over-expression of MIAT was first confirmed by quantitative PCR, approximately four- and sixfold increases were achieved in HT-3 and HeLa cells, respectively (Fig. 2a). MIAT-proficiency significantly inhibited cell proliferation in HeLa (left pane, Fig. 2b) and HT-3 cells (right pane, Fig. 2b). The inhibitory effects of MIAT in these cell lines were further confirmed with clonogenic assay. As shown in Fig. 2c, the number of formed colonies was remarkably decreased in MIAT-overexpressing cells. In addition, the anchorage-independent growth capacity was greatly compromised by ectopic MIAT expression in comparison to the control counterparts (Fig. 2d). Our data clearly demonstrated that ectopic overexpression of MIAT significantly inhibited cell proliferation and anchorage-independent growth in cervical carcinoma cell lines.

### MIAT-proficiency suppressed xenograft tumor growth in vivo

**Fig. 3:**
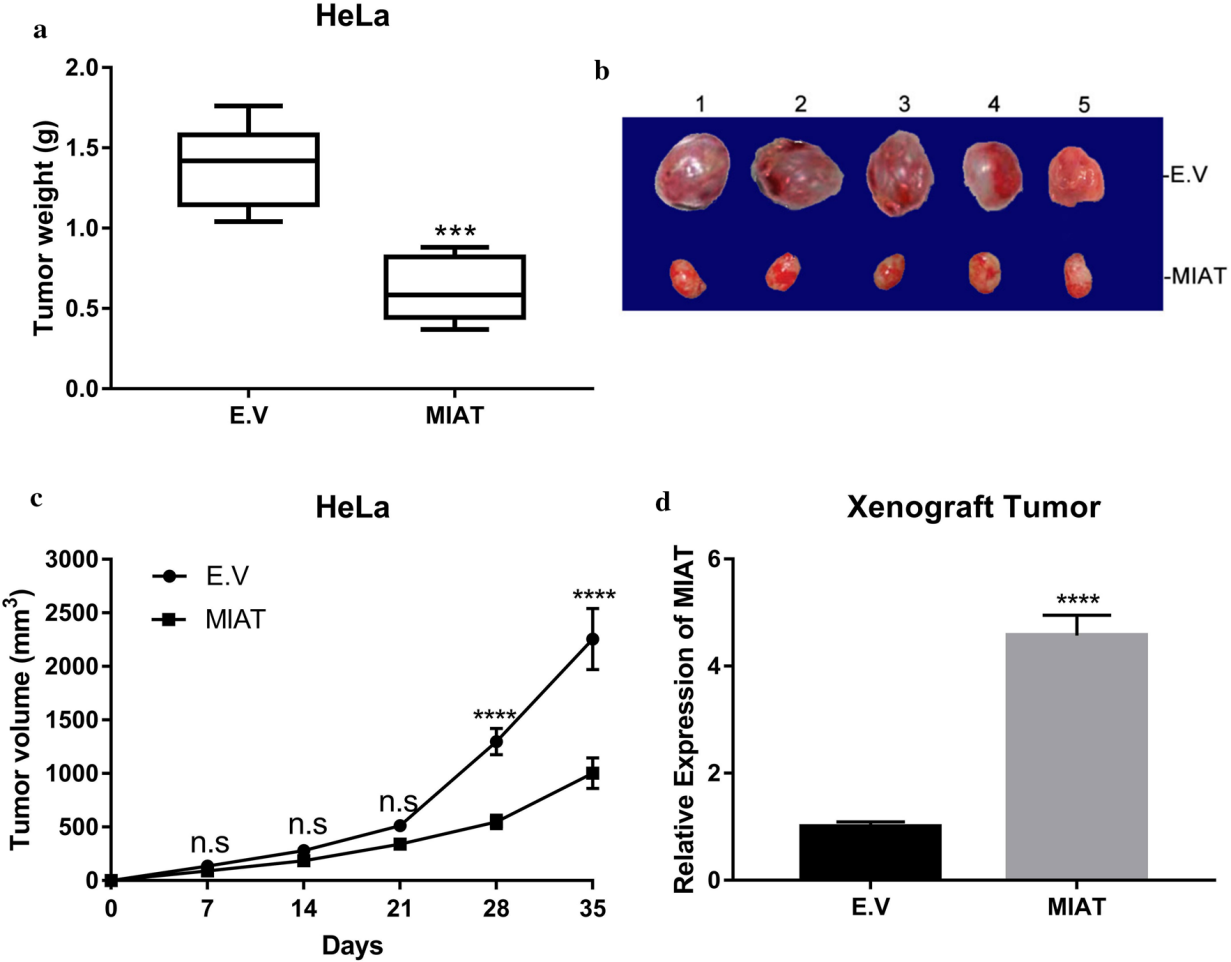
MIAT overexpression inhibited xenograft tumor growth in vivo. **a** HeLa cells transfected with either E.V or MIAT plasmids were subcutaneously inoculated into immunodeficient nude mice. The xenograft tumors were measured after sacrifice, ***p < 0.001. **b** Macroscopic images of representative xenograft tumors from both control and MIAT mice at week 5 post-inoculation. **c** The tumor growth was monitored by digital caliper at day 0, 7, 14, 21, 28 and 35 post-inoculation respectively and the volume was calculated with the formula: volume = (width)^2^ × length/2. n.s: no significance, ****p < 0.0001. **d** The persistent overexpression of MIAT in xenograft tumor was validated by real-time PCR. ****p < 0.0001

To exclude the potential artifacts associated with cell culture and confirm the tumor suppressive properties of MIAT in vivo, next we established xenograft tumor in BALB/c-nude mice. HeLa cells infected with either empty vector or MIAT-expressing lentivirus were subcutaneously inoculated into lower flanks in immunodeficient nude mice and tumor growth was continuously monitored. As shown in Fig. 3a, xenograft tumor progression was significantly inhibited by forced overexpression of MIAT, with the average weight of tumor mass was 1.38 ± 0.38 g in wild-type recipients versus 0.62 ± 0.19 g in MIAT-proficient group (Fig. 3a). The macroscopic images of xenograft tumor resected from sacrificed mice were shown in Fig. 3b. The xenograft tumor volume estimated by digital caliper demonstrated significant delay in tumor growth in MIAT-proficient mice compared to control (Fig. 3c). The persistent overexpression of MIAT in xenograft tumor was verified by real-time PCR in the sacrificed mice (Fig. 3d). Therefore, we consolidated the anti-tumoral phenotype of MIAT in cervical carcinoma xenograft tumor in vivo.

### MIAT regulated CDKN1B expression via competition with miR-150-5p

**Fig. 4:**
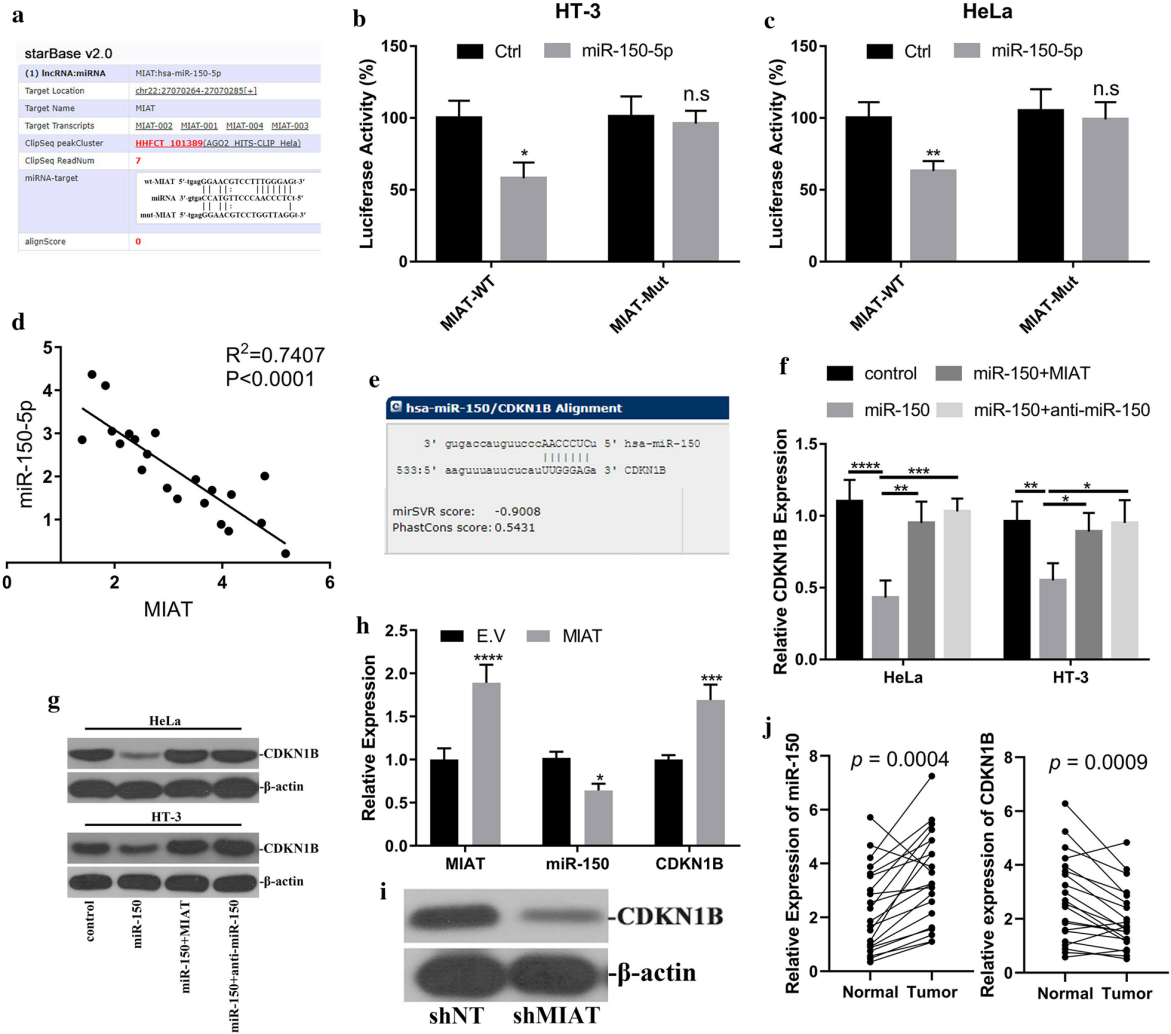
MIAT regulated CDKN1B via endogenous competition with miR-150. **a** Alignment between miR-150-5p seed region and MIAT transcript (wild-type and mutant) using Starbase online tool. **b**, **c** Luciferase reporter assay was performed to validate the regulatory effect of miR-150 on MIAT transcript. Either wild-type or putative binding site-mutant MIAT was fused to luciferase, which was co-transfected with miR-150 into HeLa and HT-3 cells. The relative luciferase activities were measured with the Bright-Glo Luciferase Assay System. n.s: no significance, *p < 0.05, **p < 0.01; **d** Correlation between miR-150 and MIAT transcripts in cervical cancer samples (n = 21). **e** Alignment between miR-150 and putative target sites in CDKN1B 3′UTR by microRNA online tool. **f** miR-150 negatively modulated CDKN1B expression, which was antagonized by MIAT. Exogenous scramble sequence, miR-150, MIAT or anti-miR-150 were transfected into HeLa (left) and HT-3 (right) cells in combination as indicated, the relative expression of CDKN1B was measured by real-time PCR in indicated cells. *p < 0.05, **p < 0.01, ***p < 0.001, ****p < 0.0001. **g** The CDKN1B protein was quantified by immunoblotting. β-actin served as loading control. **h**, **i** The MIAT-miR-150-CDKN1B axis was analyzed by q-PCR (**h**) and western blotting (**i**) in xenograft tumor. *p < 0.05, **p < 0.01, ****p < 0.0001. **j** The relative expression of both miR-150 and CDKN1B were analyzed in paired human cervical tumor samples by real-time PCR

The emerging evidences disclosed the competing endogenous (ceRNA) mode-of-action of long non-coding RNA with miRNAs [19]. Here we sought to clarify this possibility and elucidate the mechanism underlying the anti-tumor activity of MIAT. To this purpose, we employed lncRNABase online algorithm and predicted candidate miRs with the potential to directly compete with MIAT, and identified miR-150-5p at the top on the target list. The alignment between MIAT and miR-150-5p was illustrated in Fig. 4a. The regulatory effect of miR-150-5p on MIAT expression was interrogated with luciferase reporter assay. The co-transfection with miR-150 led to about 40% reduction of MIAT-fused luciferase activity in HeLa and 30% reduction in HT-3 cells, respectively (Fig. 4b). The scrambled mutation located in the putative miR-150-5p-binding site completely abrogated this inhibitory effect imposed by miR-150-5p (Fig. 4c). The correlation between relative expression of miR-150-5p and MIAT was further analyzed in cervical carcinoma tissue samples, and inverse relation was identified in Fig. 4d (R^2^ = 0.7407, *p* < 0.0001). Our data suggested that MIAT might function as molecular sponge for miR-150-5p and involve in the gene regulatory network of miR-150-5p.

Cyclin dependent kinase inhibitor 1B (CDKN1B) was an important regulator of cell cycle progression and tumor suppressor in variety of human malignancies. The previous study by Liu et al. showed that miR-150 involved in the direct regulation of CDKN1B in human prostate cancer stem cell development [20], which prompted us to investigate this possibility in cervical carcinoma. With aid of bioinformatics tools from microRNA.org, we easily identified the putative target site of miR-150-5p in 3′UTR region of CDKN1B, and alignment of corresponding sequences was illustrated in Fig. 4e. The endogenous CDKN1B expression was remarkably decreased by ectopic introduction of miR-150-5p in both HeLa and HT-3 cells (Fig. 4f). This phenotype was further confirmed at protein level as shown in Fig. 4g. However, the inhibitory effects were readily abolished by simultaneous introduction of either miR-150-5p-specific inhibitor or MIAT transcripts (Fig. 4f, g). The expression status of MIAT-miR-150-5p-CDKN1B signal axis was further characterized in xenograft tumor at both transcriptional and translational levels (Fig. 4h, i), which consolidated our in vitro observations and supported the physiological role of MIAT as ceRNA for miR-150-5p. More importantly, we demonstrated the significant up-regulation of miR-150 and down-regulation of CDKB1B in clinical cervical tumors in comparison with normal control (Fig. 4j).

### miR-150-5p-inhibition directly suppressed cervical cancer cell growth

**Fig. 5:**
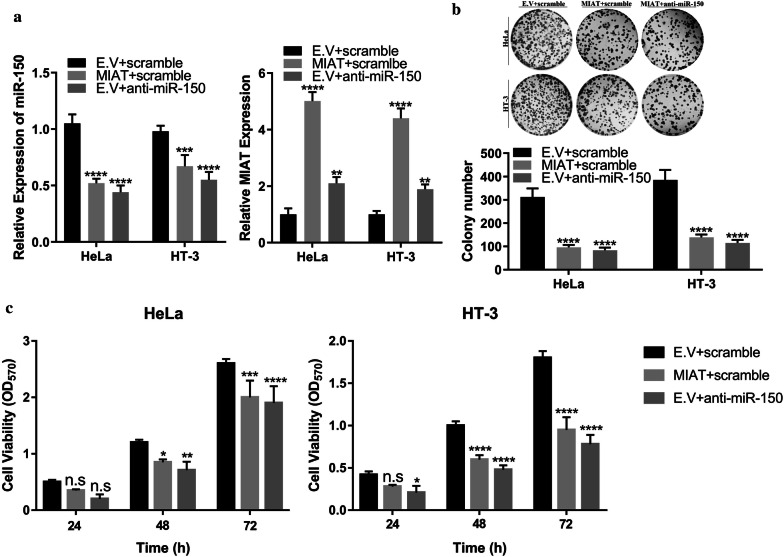
miR-150-inhibition contributed to anti-tumor activity of MIAT. **a** The relative miR-150 level (left) and MIAT (right) in response to MIAT ectopic expression or specific inhibitor was determined by real-time PCR in both HeLa and HT-3 cells. ***p < 0.001, ****p < 0.0001. **b** Colony formation assay was performed to measure the anchorage-independent growth of indicated cells. ****p < 0.0001. **c** The suppressive effect on cell proliferation of MIAT was comparable with miR-150 inhibition. The cell viability was monitor at 24, 48 and 72 h respectively using CCK-8 kit. n.s: no significance, * *p* < 0.05, ** *p* < 0.01, ***p < 0.001, ****p < 0.0001

Our previous results indicated that MIAT competitively inhibited miR-150-5p, which in turn stimulated CDKN1B expression and eventually mediated its anti-tumor function. Next, we sought to validate the pathologic role of MIAT-miR-150-5p-CDKN1B in cervical cancer via direct inhibition of miR-150-5p. The endogenous expression of miR-150-5p upon MIAT overexpression was analyzed in both HeLa and HT-3 cells. As shown in Fig. 5a, ectopic MIAT expression led to around 50% and 40% reduction of miR-150-5p in HeLa and HT-3 cells, respectively, which was comparable to the inhibitory effects conferred by miR-150-5p-specific inhibitor. The endogenous MIAT was significantly up-regulated by miR-150-5p inhibitor as well (Fig. 5a, right). Both MIAT and miR-150-5p-inhibitor significantly suppressed anchorage-independent growth in HeLa and HT-3 cells (Fig. 5b). Consistently, cell proliferation was markedly compromised in both MIAT-proficient and miR-150-5p-deficient cells (HeLa, Fig. 5c left pane; HT-3, Fig. 5c right pane). Our data unambiguously demonstrated that miR-150-5p predominately contributed to anti-tumor activity of MIAT in cervical cancer.

## Discussion

MIAT is a novel lncRNA with primary pathologic association with myocardial infarction. The emerging evidences suggested the potential involvements of MIAT in human malignancies as well. Zhang et al. [9] demonstrated that MIAT regulated zinc finger E-box binding homeobox 1 expression by sponging miR-150 and promoted cell invasion in non-small-cell lung cancer. The study performed by Li et al. [10] highlighted the involvement of MIAT/miR-29a-3p/HDAC4 axis in regulation of cell biological behaviors and development of gastric cancer. Crea et al. [11] also provided preliminary evidences indicating that MIAT was selectively upregulated in neuroendocrine prostate cancer and highly likely interacted with polycomb genes. On the contrary, tumor suppressive property of MIAT was proposed by Zhang et al. [21] in glioblastoma multiforme, wherein expression of MIAT, PART1, MGC21881, GAS5 and PAR5 associated with favorable clinical outcomes. Consistent with this, we first characterized aberrant low-level of MIAT in cervical carcinoma both in vivo and in vitro. High expression of MIAT significantly associated with better prognosis in cervical cancer patients. Ectopic expression of MIAT in cervical cancer cells remarkably suppressed cell proliferation, anchorage-independent growth. The in vitro phenotype was further consolidated in vivo using xenograft tumor mice model. Mechanistically, we elucidated that MIAT functioned as ceRNA against miR-150-5p, and MIAT abundance inversely correlated to endogenous miR-150-5p in cervical carcinomas. Notably, we identified CDKN1B as direct target of miR-150-5p, and the negative regulation of CDKN1B by miR-150-5p was readily antagonized by co-introduction of MIAT. The predominant role of miR-150-5p in mediating the anti-tumor properties of MIAT was consolidated by directly introducing either miR-150-5p-specific inhibitor or MIAT. Our study for the first time highlighted the critical role of MIAT-miR-150-5p-CDKN1B axis in cervical carcinoma. Noting worthily, the molecular mechanism underlying low-expression of MIAT in cervical carcinoma was still to be investigated in future.

The CDKN1B was an important regulator of cell cycle progression and critically involved in G1 arrest [22]. CDKN1B physiologically complexed with cyclin E-CDK2 or cyclin D-CDK4 to hold cells at quiescent state. Assembling evidences suggested the intimate association between deficiencies of CDKN1B with array of human cancers. For instance, case–control study conducted by Xiang et al. [23] uncovered the association of CDKN1B gene polymorphisms with susceptibility to breast cancer, which was further supported by Canbay et al. [24] and Landa et al. [25]. Sirma et al. [26] identified that loss of CDKN1B/p27Kip1 expression was associated with ERG fusion-negative prostate cancer but not the total patient prognosis. Lynch et al. [27] reported the tumor suppressor role of miR-24 in prostate cancer cells via direct regulation of CDKN1B. Here we shed light on the previously unrecognized molecular mechanism underlying aberrant down-regulation of CDKN1B in cervical carcinoma and uncovered the MIAT-miR-150-5p-CDKN1B signaling pathway in this disease. Consistent with previous study that suggested miR-150 negatively and post-transcriptionally regulated CDKN1B in prostate cancer stem cells, here we consolidated this regulatory axis in cervical carcinoma which was subjected to MIAT competition as well.

The contradictory roles of miR-150-5p in human cancers have been concluded so far, which might attribute to disease context. A number of investigations were in support of the anti-tumor properties of miR-150. Koshizuka et al. [28] demonstrated that miR-150-5p and miR-150-3p inhibited cancer cell aggressiveness by targeting SPOCK1 in head and neck squamous cell carcinoma. Fan et al. [29] reported that miR-150 alleviated epithelial-mesenchymal transition and cell invasion of colorectal cancer through targeting Gli1. In the paclitaxel-resistant ovarian cancer cells, Kim et al. [30] showed that miR-150 enhanced apoptotic and anti-tumor effects of paclitaxel. In thyroid cancer, Bai et al. [31] reported that miR-150 inhibited cell growth in vitro and in vivo by restraining the RAB11A/WNT/β-Catenin pathway. Intriguingly, Zhang et al. [9] demonstrated that MIAT regulated zinc finger E-box binding homeobox 1 expression by sponging miR-150 and promoting cell invasion in non-small-cell lung cancer. In contrast, the oncogenic properties of miR-150 have also been proposed by array of studies. For instance, Li et al. [32] demonstrated that miR-150 promoted cellular metastasis in non-small-cell lung cancer by targeting FOXO4. Likewise, Zhao et al. [33] reported that miR-150 promoted the cell invasion of prostate cancer cells by directly regulating the expression of p53. The circulating level miR-150 along with miR-34a have been proposed associating with colorectal cancer progression [34]. Huang et al. [35] demonstrated that miR-150 promoted human breast cancer growth and malignant behavior by targeting the pro-apoptotic purinergic P2X7 receptor. However, the expression status of miR-150-5p and potential mechanistic involvements in human cervical carcinoma has not been fully uncovered. Here we characterized the up-regulation of miR-150-5p in cervical carcinoma, and ectopic expression of either MIAT or its specific inhibitor significantly suppressed the cell malignant behavior, which was in support of an oncogenic role of miR-150-5p in this disease.

## Conclusion

In summary, here we identified the anti-tumor property of MIAT through endogenously competing for miR-150 in regulation of CDKN1B, which consequently contributed to our comprehensive understanding of mechanistic involvements of long non-coding RNA in cervical carcinoma. Importantly, our results uncovered the potential therapeutic value of miR-150-5p-specific inhibitor in MIAT-negative cervical cancer.

## Data Availability

All data generated or analyzed during this study are included in this published article.
